# Prediction of lncRNA and disease associations based on residual graph convolutional networks with attention mechanism

**DOI:** 10.1038/s41598-024-55957-y

**Published:** 2024-03-02

**Authors:** Shengchang Wang, Jiaqing Qiao, Shou Feng

**Affiliations:** 1 School of Electronic and Information Engineering, Harbin Institute of Technology, Harbin, 150001 China; 2 College of Information and Communication Engineering, Harbin Engineering University, Harbin, 150001 China

**Keywords:** LncRNA-disease associations, Similarity, Graph convolutional networks, Residual network, Attention mechanism, Data mining, Classification and taxonomy, Machine learning, Gene ontology

## Abstract

LncRNAs are non-coding RNAs with a length of more than 200 nucleotides. More and more evidence shows that lncRNAs are inextricably linked with diseases. To make up for the shortcomings of traditional methods, researchers began to collect relevant biological data in the database and used bioinformatics prediction tools to predict the associations between lncRNAs and diseases, which greatly improved the efficiency of the study. To improve the prediction accuracy of current methods, we propose a new lncRNA-disease associations prediction method with attention mechanism, called ResGCN-A. Firstly, we integrated lncRNA functional similarity, lncRNA Gaussian interaction profile kernel similarity, disease semantic similarity, and disease Gaussian interaction profile kernel similarity to obtain lncRNA comprehensive similarity and disease comprehensive similarity. Secondly, the residual graph convolutional network was used to extract the local features of lncRNAs and diseases. Thirdly, the new attention mechanism was used to assign the weight of the above features to further obtain the potential features of lncRNAs and diseases. Finally, the training set required by the Extra-Trees classifier was obtained by concatenating potential features, and the potential associations between lncRNAs and diseases were obtained by the trained Extra-Trees classifier. ResGCN-A combines the residual graph convolutional network with the attention mechanism to realize the local and global features fusion of lncRNA and diseases, which is beneficial to obtain more accurate features and improve the prediction accuracy. In the experiment, ResGCN-A was compared with five other methods through 5-fold cross-validation. The results show that the AUC value and AUPR value obtained by ResGCN-A are 0.9916 and 0.9951, which are superior to the other five methods. In addition, case studies and robustness evaluation have shown that ResGCN-A is an effective method for predicting lncRNA-disease associations. The source code for ResGCN-A will be available at https://github.com/Wangxiuxiun/ResGCN-A.

## Introduction

Long non-coding RNA (lncRNA) is a class of non-coding RNA with more than 200 nucleotides. LncRNAs were originally thought to be the “noise” of the body’s metabolic activity, which has no biological function^1^. However, more and more evidence shows that lncRNAs are closely related to many life activities in the human body, and they can participate in dosage compensation, epigenetic regulation, transcription regulation, post-transcription regulation, cell cycle regulation, cell differentiation regulation, and other life activities^2^. In addition, changes in many lncRNAs have been linked to complex diseases, including cancer and neurological diseases. The abnormal expression of lncRNA, such as mutation, up-regulation, or down-regulation, leads to the generation of diseases^3,4^. If the diseases associated with abnormal lncRNAs can be known, the treatment plan for the diseases can be proposed in advance, and the cure level of the diseases will also be improved. Therefore, it is necessary to study the relationship between lncRNAs and diseases. However, at present, traditional experimental methods such as in situ hybridization and over-expression techniques are mainly used to study lncRNAs. Although the results of such traditional methods are accurate, their efficiency is too low. The use of computational methods to predict the lncRNA-disease associations helps save the time and cost of traditional research methods, which can greatly improve the efficiency of research. Currently, existing computational methods for predicting lncRNA-disease associations can be roughly divided into three categories: biological network-based methods, machine learning-based methods, and other methods.

Biological network-based methods are based on the hypothesis that lncRNAs with similar functions may be associated with diseases with similar phenotypes. Sun et al.^5^ proposed a computational model of global networks, RWRlncD, based on functionally similar networks of lncRNAs. In this method, lncRNA-disease associations networks, disease similarity networks, and functional similarity networks of lncRNAs were first constructed, and then random walk restarts were used to predict potential lncRNA-disease associations on functional similarity networks of lncRNAs. Xi et al.^6^ proposed a collaborative matrix decomposition method LDCMFC based on correlation coefficients. This method replaces the traditional minimization of Euclidean distance with the maximization of entropy, which improves the robustness of the algorithm. Wang et al.^7^ proposed LDAP-WMPS based on weight matrix and projection scoring. It obtained the predicted association matrix by proportionally merging the projected disease score and the projected lncRNA score. Xie et al.^8^ proposed a prediction method SSMF-BLNP that combined selective similarity matrix fusion with bidirectional linear neighborhood label propagation, proposed new lncRNA similarity and disease similarity, and better solved the problems of noise and self-similarity loss in existing similarity integration methods. Lin et al.^9^ proposed a method based on probabilistic matrix decomposition, SCCPMD. In this method, the microbe-disease association information is added, and the matrix decomposition is constrained by the similarity matrix. In addition, the noise effect in the similarity matrix is eliminated by the logistic correction method, which improves the prediction accuracy.

The machine learning-based methods train the classifier based on the features of lncRNAs associated with known diseases and unknown lncRNAs, and rank candidate lncRNAs based on the differences in biological features between the sets. Wu et al.^10^ proposed GAMCLDA, a prediction method based on graph autoencoder matrix completion. In this method, the graph convolutional network was first used to obtain the potential factor vectors of lncRNA and disease from the local graph structure and features of nodes, and then the inner product of the two was used to reconstruct the lncRNA-disease association matrix to achieve prediction. Wu et al.^11^ proposed an Extra-Trees prediction method MLGCNET based on multi-layer graph embedding aggregation. This method adopted the top-k method to reconstruct the similarity, used the multi-layer graph convolutional network for feature extraction and fusion, and finally used the Extra-Trees classifier for prediction. Lan et al.^12^ proposed a method based on graph attention network, GANLDA. GANLDA obtained biological node features of lncRNAs from the lncRNA-miRNA associations, the lncRNA-GO associations, and the lncRNA-Gene associations, and disease node features from the disease-miRNA associations and disease-gene associations. Firstly, the principal component analysis method was used to denoise the features of the two biological nodes, then the graph attention network was used to learn the potential feature representation of the lncRNA and the disease, and finally, the multilayer perceptron was used to predict the associations between the lncRNA and the disease. However, the network depth of this method is low and the feature information of the neighbor node is not fully utilized. Liang et al.^13^ proposed MAGCNSE. MAGCNSE used graph convolutional networks to obtain multiple feature matrices from multi-view similarity graphs of lncRNAs and diseases. However, this method only used lncRNAs and diseases information and did not use other biological information, such as miRNAs, proteins and drugs. Zhao et al.^14^ proposed HGATLDA, a meta-path-based heterogeneous graph attention network, which has improved its ability to fuse node features, heterogeneous topological structures and semantic information. In this method, the K-nearest neighbor graph method is used to integrate the similarity information, and node embedding of graph attention network learning features is used, then subgraph attention network based on meta-path is used to aggregate the embedding of each subgraph. Finally, neural inductive matrix completion is used to reconstruct the lncRNA-disease associations. Zhang et al.^15^ proposed CapsNet-LDA, A prediction method of attention mechanism and capsule network based on multi-perspective data. In this method, stacking autoencoders and attention mechanisms were used to integrate similarity networks to obtain lncRNA-disease associations network A. The capsule network with BiLSTM was used to process the associations network A and get the prediction result. Zhang et al.^16^ proposed a method based on graph representation learning, LDAGRL, which integrated multiple data associations into a bridge-like heterogeneous information network, used structural deep network embedding to learn node embedding, and finally used XGBoost classifier to predict the potential associations between lncRNAs and diseases.

In addition to these two categories of methods, some methods based on other theories or strategies have been proposed. Due to the limited number of experimentally validated lncRNA-disease associations, researchers have turned to predicting lncRNA-disease associations based on known disease-related genes, miRNAs, and lncRNAs in relation to genes or miRNAs. Liu et al.^17^ developed a prediction method that did not rely on known lncRNA-disease associations, but instead predicted potential human lncRNA-disease associations by integrating known human disease genes and human lncRNAs with gene expression profiles. The method divided lncRNAs into tissue-specific and non-tissue-specific components. First, a tissue-specific score was calculated based on the expression levels of all lncRNAs in different tissues, and then possible associations between tissue-specific lncRNAs and human diseases were predicted. However, the method could not predict disease-associated lncRNAs without relevant gene records. Chen et al.^18^ proposed the HGLDA model based on the hypergeometric distribution in statistical methods. This model integrates disease semantic similarity, miRNA-disease associations, and miRNA-lncRNA interactions to obtain functional similarity of lncRNAs. Finally, the model tested the hypergeometric distribution of each lncRNA-disease pair by testing whether the lncRNA-disease pair significantly shared miRNA interacting with the two.

Therefore, although most of the existing methods can successfully predict the potential lncRNA-disease associations, there is still room for improvement. Firstly, the known lncRNA-disease associations number is too small, resulting in the obtained lncRNA-disease associations matrix being very sparse, which leads to the imbalance of positive and negative samples, and limits the prediction performance of the methods. Secondly, the lack of global information on lncRNA and disease features extracted by most methods leads to inaccurate features, which limits the prediction accuracy of the methods. Moreover, the long-running time also limits the prediction efficiency of the methods. To ameliorate these problems, we applied the computational framework of the method GAERF^25^ and imported the residual method and attention mechanism in feature extraction, to propose a new lncRNA-disease associations prediction method based on residual graph convolutional network with attention mechanism (ResGCN-A).

In this method, the residual graph convolutional network combined with the attention mechanism is used to obtain the deep features of lncRNA and disease. The obtained deep features not only pay attention to the local information of the original features, but also include the global information of the original features. The experimental results show that our prediction method is superior to the other five methods. The main contributions of this paper are given as follows:

- A novel lncRNA-disease associations prediction method is proposed, namely, the residual graph convolutional network based on attention mechanism ResGCN-A. The residual graph convolutional network is a combination of the residual network block and the graph convolutional network, which can extract the local features of lncRNAs and diseases. The attention mechanism module is used to obtain the global information of lncRNAs and diseases. Then, the combination of residual graph convolutional network block and attention mechanism was adopted to realize the fusion of local and global features of lncRNAs and diseases, which made the potential features of the two obtained more accurate and improved the prediction efficiency.
- A feature extraction module based on residual network and graph convolutional network is designed, that is, residual graph convolutional network block. The block consists of two graph convolutional networks. The local features extracted by the two graph convolutional networks are combined in the form of residuals, which preserves the intermediate features between the layers of the graph network, enriches the feature hierarchy, and helps to obtain more accurate local information. At the same time, the import of residual network can prevent the problem of gradient explosion in the learning process of the model and improve the model stability.
- An attention mechanism based on the two-dimensional matrix is designed to assign different attention weights to features. Specifically, the attention mechanism consists of three fully connected layers and a row summation module. The full-connection layer maps the features, and the row summation module summarizes the mapped features horizontally. The sum of each row is the attention weight coefficient of each row’s feature. This attention mechanism can avoid the operation of matrix dimension transformation and reduce the computational cost of the model. Moreover, the attention mechanism verification shows that the attention mechanism can improve prediction accuracy.

Based on these contributions, ResGCN-A obtained an AUC value of 0.9916 and an AUPR value of 0.9951 in the 5-fold cross-validation. In addition, case studies of prostate cancer and colon cancer have shown that ResGCN-A has a good predictive performance.

## Methods

### Method overview

**Figure 1:**
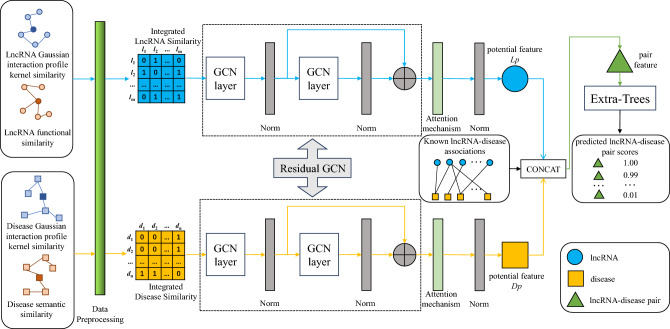
The flowchart of ResGCN-A.

The flowchart of the ResGCN-A is shown in Fig. 1, which can be divided into five steps. The first step is to construct the similarity feature matrices, which include the lncRNA similarity feature matrix and the disease similarity feature matrix. The lncRNA similarity feature matrix is a combination of the lncRNA Gaussian interaction profile kernel similarity matrix and the lncRNA functional similarity matrix. The disease similarity feature matrix is composed of the disease Gaussian interaction profile kernel similarity matrix and the disease semantic similarity matrix. The second step is to use the graph convolutional network in the residual form to extract the local features of lncRNA and disease. The third step is to use the attention mechanism to obtain global information of lncRNA features and disease features. After batch normalization, the potential features of the two will be obtained. The fourth step is to concatenate the potential features of lncRNA and the potential features of disease together to form the features of association pairs for training the Extra-Trees classifier. In the last step, the trained classifier is used to predict potential lncRNA-disease associations.

### Data collection

LncRNA-disease associations are collected from LncRNADisease^19^, Lnc2Cancer^20^ and GeneRIF^21^. After preprocessing the collected data, 2588 associations of 228 lncRNAs and 403 diseases were obtained, which constituted the lncRNA-disease associations matrix *A* with a size of 228 $\times$ 403. In matrix A, if a lncRNA has been confirmed to be associated with a disease, the cross position of the two is filled with 1. On the contrary, If there is no confirmed association between the two, the cross position of the two is filled with 0.

### Computing lncRNA similarity and disease similarity

This part first introduces the calculation methods of four similarity matrices, including the lncRNA Gaussian interaction profile kernel similarity matrix, the lncRNA functional similarity matrix, the disease Gaussian interaction profile kernel similarity matrix and the disease semantic similarity matrix, and then introduces the integration method of lncRNA similarity feature matrix and disease similarity feature matrix.

#### LncRNA Gaussian interaction profile kernel similarity

Based on the previous method^22^, the lncRNA Gaussian interaction profile kernel similarity matrix (*lncG*) is calculated as:

1 $$\begin{aligned} lncG(l_{i}, l_{j})= & {} exp(-\gamma _{l}||A(i,:)-A(j,:)||^2) \end{aligned}$$

2 $$\begin{aligned} \gamma _{l}= & {} 1/\left( \frac{1}{M}\sum _{m=1}^{M}||A(m,:)||^2\right) \end{aligned}$$

where A represents the lncRNA-disease associations matrix of size $M\times N$, *M* is the number of lncRNAs, and *N* is the number of diseases. $\gamma$ is a hyperparameter in the Gaussian kernel.

#### Disease Gaussian interaction profile kernel similarity

Similarly, the disease Gaussian interaction profile kernel similarity matrix (*disG*) is calculated as:

3 $$\begin{aligned} disG(d_{i}, d_{j})= & {} exp(-\gamma _{d}||A(:, i)-A(:, j)||^2) \end{aligned}$$

4 $$\begin{aligned} \gamma _{d}= & {} 1/\left( \frac{1}{N}\sum _{n=1}^{N}||A(:, n)||^2\right) \end{aligned}$$

where A represents the lncRNA-disease associations matrix of size $M\times N$, *M* is the number of lncRNAs, and *N* is the number of diseases. $\gamma$ is a hyperparameter in the Gaussian kernel.

#### Disease semantic similarity

Referring to previous work^23^, the disease semantic similarity (*disS*) is calculated according to the disease DAG. The disease DAG is constructed by mapping the disease DOIDs into the MeSH. Taking disease $d_n$ for example, DAG$(d_{n})=\{V_{d_{n}}, E_{d_{n}}\}$. $V_{d_{n}}$ represents the set of disease $d_n$ and its ancestor diseases, and $E_{d_{n}}$ represents the set of edges between the diseases in $V_{d_{n}}$. For any disease *n* in the set $V_{d_{n}}$, the contribution of *n* to the disease $d_{n}$ can be calculated as:

5 $$\begin{aligned} D_{d_{n}}(n)={ {\left\{ \begin{array}{ll} 1,&{} {if\ n=d_{n}}\\ max(\alpha \times D_{d_n}(n')|n'\in C_{n}),&{} {otherwise} \end{array}\right. }} \end{aligned}$$

where, $\alpha$ is the semantic contribution factor of the edge between *n* and $n'$ in set $E_{dn}$, which is set to 0.5. $C_{n}$ represents the set of children with disease *n*.

The semantic value (*disV*) of disease $d_n$ is defined as:

6 $$\begin{aligned} disV(d_n)=\sum _{n\in V_{d_n}}^{}D_{d_n}(n) \end{aligned}$$

Finally, the semantic similarity of the two diseases, $d_{i}$ and $d_{j}$, is defined as:

7 $$\begin{aligned} disS(d_{i}, d_{j})=\frac{\sum _{n\in V_{d_{i}}\cap V_{d_{j}}}^{}(D_{d_{i}}(n)+D_{d_{j}}(n))}{disV(d_{i})+disV(d_{j})} \end{aligned}$$

where $D_{d_{i}}(n)$ is the semantic value of *n* related to disease $d_{i}$, $D_{d_{j}}(n)$ is the semantic value of n related to disease $d_{j}$, $disV(d_{i})$ is the semantic value of disease $d_{i}$, $disV(d_{j})$ is the semantic value of disease $d_{j}$. In this way, we can obtain the disease semantic similarity matrix.

For convenience, the function doSim in the DOSE package^24^ is used to process the DOIDs of the input diseases, and the disease semantic similarity matrix *disS* can be obtained by using the measurement method of “Wang” in function doSim.

#### LncRNA functional similarity

Based on existing calculation methods^5^, given lncRNA $l_i$ associated with $n_1$ diseases and lncRNA $l_j$ associated with $n_2$ diseases, their functional similarity value is defined as:

8 $$\begin{aligned} lncF(l_i, l_j)= & {} \frac{\sum \nolimits _{d\in D(l_j)}dS(d, D(l_i))+\sum \nolimits _{d\in D(l_i)}dS(d, D(l_j))}{n_1+n_2} \end{aligned}$$

9 $$\begin{aligned} dS(d, D(l_i))= & {} \max \limits _{d_1\in D(l_i)}(disS(d, d_1)) \end{aligned}$$

where $D(l_i)$ represents the set of $n_1$ diseases related to lncRNA $l_i$, and $D(l_j)$ represents the set of $n_2$ diseases related to lncRNA $l_j$.

In this way, we can obtain the lncRNA functional similarity matrix *lncF*.

#### Integration of similarity matrix

*lncG* and *lncF* are integrated into the lncRNA similarity matrix, which is referred to as *lncSM* for short. Firstly, the preliminary lncRNA similarity matrix is defined as:

10 $$\begin{aligned} plncSM=\frac{lncG+lncF}{2} \end{aligned}$$

Then, by referring to the data processing method in GAERF method^25^, we sorted the similarity values between any lncRNA and all lncRNAs in the matrix, and modified the value of the first *kl* similarity values in the matrix to 1, and the remaining positions to 0. In this way, we can obtain the integrated lncRNA similarity matrix *lncSM*.

Similarly, parameter *kd*, corresponding to parameter *kl*, was used to integrate *disG* and *disS*, and the integrated disease similarity matrix (disSM for short) was obtained.

### Residual graph convolutional networks

**Figure 2:**
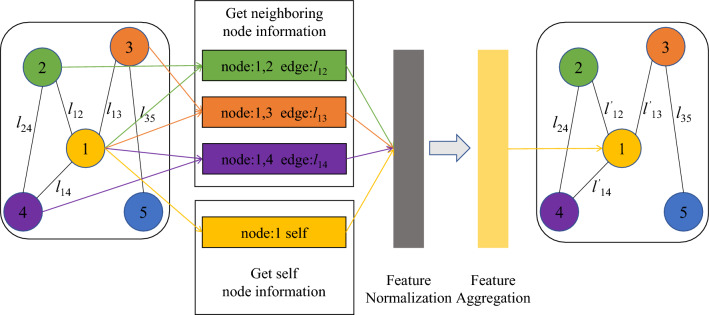
The flowchart of GCN.

Graph convolutional network is mainly used for feature extraction. It combines the features of the node itself with those of its adjacent nodes to obtain the deep features of the original node. As shown in Fig. 2, for a node, GCN first obtains the features of the node itself and the features of adjacent nodes. Then the obtained features are normalized and aggregated. After aggregation, a new node feature is obtained, which includes not only the original node features, but also the original node’s adjacent node features.

Assuming that the input feature is $X^{(0)}$, the feature $X^{(1)}$after one layer of GCN processing is:

11 $$\begin{aligned} X^{(1)}=relu(\hat{D}^{-\frac{1}{2}}\hat{B}\hat{D}^{-\frac{1}{2}}X^{(0)}\Theta ) \end{aligned}$$

where *relu* is the relu activation function, $\hat{B}$ is the matrix after adding the loop to *B*, *B* is the adjacency matrix representing the relationship between each node, $\hat{D}$ is the degree matrix of $\hat{B}$, *X* is the feature matrix, $\Theta$ is the weight matrix in the training process.

**Figure 3:**
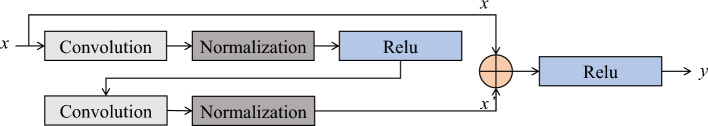
The flowchart of the residual block.

The residual network can effectively reduce the training error. The residual block network in general form is shown in Fig. 3. The residual block network is composed of convolution layer, normalization layer, activation layer and summation layer. Assuming that the input feature is *x*, in the residual network, it passes through the convolutional layer, the normalization layer, the activation layer, the convolutional layer and the normalization layer in turn, and finally the feature $x'$ will be obtained. After summing *x* and $x'$ and passing through the activation layer, the feature *y* is obtained.

In the model proposed in this paper, the convolutional layer in the residual-block network is replaced by the graph convolutional network, and the input of the summation layer is replaced by the output of the first layer GCN and the output of the second layer GCN, to obtain the residual GCN in Fig. 1.

Given the integrated similarity lncSM of lncRNA, we can use the residual graph convolutional network to obtain the local features of lncRNA:

12 $$\begin{aligned} lncSM\_local=relu(relu(norm(lncSM^{(1)}))+norm(lncSM^{(2)})) \end{aligned}$$

where *relu* is the relu activation function, *norm* is the normalization of features, $lncSM^{(1)}$ is the features of *lncSM* after one graph convolutional network and $lncSM^{(2)}$ is the features of *lncSM* after two graph convolutional networks. In the same way, we can also obtain the local features of the disease $disSM\_local$.

### Attention mechanism

The attention mechanism in neural networks is a method to allocate computing resources to more important tasks and solve the problem of information overload in the case of limited computing power. Since the features extracted by the residual graph convolutional network only contain local information, to realize the fusion of global information and local information, we can use the attention mechanism to extract the global information of features. An important step in the attention mechanism is the calculation of attention value. The calculation steps of attention value are as follows: firstly, the attention distribution is calculated on all the input information; Then, input information is weighted according to the distribution of attention. Models with attentional mechanisms can pay more attention to key input features when attention resources are limited, thus improving the efficiency of neural networks.

**Figure 4:**
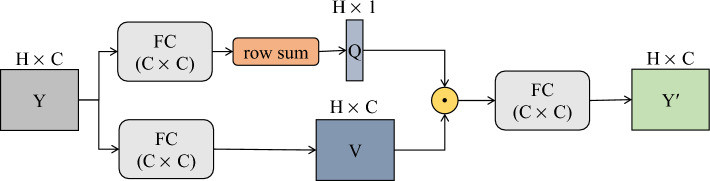
The flowchart of the attention mechanism.

In this paper, an attention mechanism for processing the two-dimensional matrix is proposed to obtain global information of lncRNA features and disease features. The flowchart of the attention mechanism is shown in Fig. 4. For the input deep feature *Y* with the size of $H\times C$, it is firstly passed through two fully connected layers to obtain feature $Q'$ with the size of $H\times C$ and feature *V* with the size of $H\times C$ respectively. Then, feature $Q'$ is summed over each row to obtain the attention weight distribution matrix *Q* of size $H\times 1$. Furthermore, the attention weight distribution matrix *Q* is multiplied by feature *V*, and the result is passed through a fully connected layer to obtain the feature $Y'$ after attention allocation. For the input feature *Y* of size $H\times C$, the formula for the whole process is:

13 $$\begin{aligned} Q= & {} rs(Y\cdot W_q+b_q) \end{aligned}$$

14 $$\begin{aligned} V= & {} Y\cdot W_v+b_v \end{aligned}$$

15 $$\begin{aligned} Y'= & {} (Q\cdot V)\cdot W+b \end{aligned}$$

where $W_q$, $W_v$ and *W* all represent the weight matrix with the size of $C\times C$; $b_q$, $b_v$, and *b* all represent bias matrices of size $H\times C$; and $rs(\cdot )$ represents row summation operations.

Given the local feature $lncSM\_local$ of lncRNA, we use the attention mechanism to process it globally and obtain the potential feature *Lp* of lncRNA:

16 $$\begin{aligned} Q_l= & {} rs(lncSM\_local\cdot W_{ql}+b_{ql}) \end{aligned}$$

17 $$\begin{aligned} V_l= & {} lncSM\_local\cdot W_{vl}+b_{vl} \end{aligned}$$

18 $$\begin{aligned} Lp= & {} (Q_l\cdot V_l)\cdot W_l+b_l \end{aligned}$$

In this way, we can obtain the potential feature *Lp* of lncRNA. Similarly, the potential feature of the disease *Dp* , can also be obtained.

### Loss function

We used the binary cross-entropy loss function during the calculation of lncRNA potential features and disease potential features:

19 $$\begin{aligned} \ell oss= & {} \frac{1}{S}\sum _{i,j}^{}(A_{ij}log(\hat{A}_{ij})+(1-A_{ij})log(1-\hat{A}_{ij})) \end{aligned}$$

20 $$\begin{aligned} \hat{A}= & {} sigmoid(Lp\cdot temp\cdot Dp^T) \end{aligned}$$

where *S* is the size of data *A* and $\hat{A}$, with a value equal to $M\times N$, *i* ranges from 0 to $M-1$, and *j* ranges from 0 to $N-1$, $sigmoid(\cdot )$ is the activation function, *Lp* is the potential features of lncRNA with size $M\times C$, *Dp* is the potential features of disease with size $N\times C$, *temp* is the trainable parameter matrix with size $C\times C$.

### Extra-Trees

Extra-Trees, full name extremely randomized trees, is a tree-based integration method commonly used to solve supervised classification and regression problems in deep learning^26^. LncRNA-disease associations prediction problem is actually a classification problem. LncRNAs associated with diseases are classified as positive pairs, while those not associated with diseases are classified as negative pairs. So we chose an Extra-Trees classifier to predict lncRNA-disease associations.

We firstly concatenated the features *Lp* and *Dp* to get the features of each lncRNA-disease pair. For example, the feature of the lncRNA-disease pair ($l_i$,$d_j$) is:

21 $$\begin{aligned} \begin{aligned} Feature(l_i,d_j)&=[Lp(l_i);Dp(d_j)]\\&=[lp(i,1),lp(i,2),...,lp(i,C); dp(j,1), dp(j,2),..., dp(j,C)] \end{aligned} \end{aligned}$$

where *lp*(*i*, 1) represents the 1st element of row *i* in the matrix *Lp*, and so on. Then, the known associations matrix *A* is used as labels and used together with the pair features to train the Extra-Trees classifier. Finally, the trained Extra-Trees classifier was used to predict the unknown lncRNA-disease pairs. The higher the prediction score, the more possible the lncRNA in the pair is to be associated with the disease.

## Results

### Evaluation criteria

The method evaluation criteria used in this experiment include the ROC curve (receiver operating characteristic curve), AUC value (the area under curve) and PR curve (precision-recall curve), AUPR (the area under precision-recall curve).

Prediction of lncRNA-disease associations is essentially a binary classification. LncRNAs associated with the disease are classified as positive, while those without are classified as negative. In the prediction model, a score matrix of the associations between each lncRNA and each disease can be obtained. We can select a threshold value according to this score value. If the score exceeds this threshold, it will be judged as a positive sample, while if the score is lower than this threshold, it will be judged as a negative sample. By comparing the predicted results of the model with the known sample associations before the prediction, four types of samples can be obtained, namely, positive samples predicted by the model as positive(TP), negative samples predicted by the model as positive(FP), positive samples predicted by the model as negative(FN), and negative samples predicted by the model as negative(TN).

By selecting different thresholds, the quantity of TP, FP, FN and TN under different thresholds can be obtained, thus the true positive rate (TPR) and the false positive rate(FPR) can be calculated. With FPR as the horizontal axis and TPR as the vertical axis, we can obtain an ROC curve. The calculation of TPR and FPR is shown as:

22 $$\begin{aligned} TPR= & {} \frac{TP}{TP+FN} \end{aligned}$$

23 $$\begin{aligned} FPR= & {} \frac{FP}{FP+TN} \end{aligned}$$

The AUC value is the area under the ROC curve. Usually, the AUC value is greater than 0 and less than 1. The closer the ROC curve is to the upper left, the closer the AUC value is to 1, and the better the model performance will be. Therefore, the performance of the model can be judged by comparing the AUC value of each model. The larger the AUC value is, the better the performance of the model is.

Similar to the above TPR and FPR, the corresponding recall and precision can be calculated by selecting different thresholds. A PR curve can be obtained by taking recall as the horizontal axis and precision as the vertical axis. The calculation of recall and precision is shown as:

24 $$\begin{aligned} Recall= & {} \frac{TP}{TP+FN} \end{aligned}$$

25 $$\begin{aligned} Precision= & {} \frac{TP}{FP+TP} \end{aligned}$$

The AUPR value is the area under the PR curve, and the AUPR value is between 0 and 1. The larger the AUPR value is, the better the model performance is.

Based on the evaluation criteria, the performance of the method is evaluated by using 5-fold cross-validation. In the process of validation, to reduce the impact of raw data imbalance on model evaluation, we randomly selected unlabeled samples with the same number of positive samples as negative samples. Then the positive and negative samples are divided into five parts, one of which is used as a test set, and the other four parts are used as a training set to train the classifier and finally obtain the validation result. To reduce the randomness of the experiment, we repeated the experiment 20 times for each validation and took the average of the results of these 20 experiments as the final validation result.

### Parameter setting

**Figure 5:**
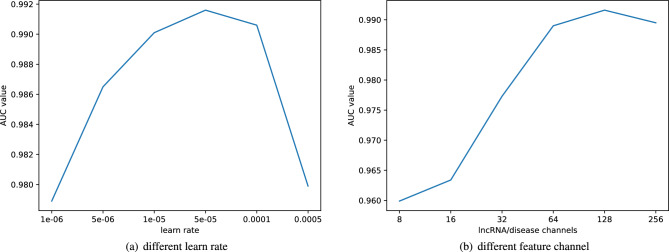
The AUC value of different learn rate and different feature channels.

Many parameters need to be set in advance in the deep learning model. The optimization algorithm adopted by ResGCN-A is *Adam*. The random number seed *n* of the generated weight matrix is set to 1 and the train *epoch* is set to 1000. The control variable method is used to select the learning rate *lr*, the feature dimension *C* of lncRNA and disease, and the parameter *kl* when integrating the similarity matrix of lncRNA and *kd* when integrating the similarity matrix of disease. The effect of the model is referred to as the AUC obtained by the 5-fold cross-validation method. The learning rate *lr* is selected from [0.000001, 0.000005, 0.00001, 0.00005, 0.0001, 0.0005] and the result is shown in Fig. 5a. The parameter *C* is selected from [8, 16, 32, 64, 128, 256], and the result is shown in Fig. 5b. It can be observed in Fig. 5a that ResGCN-A works best when the learning rate is set to 0.00005. Figure 5b shows that when the feature dimension *C* of lncRNA and disease is set to 128, ResGCN-A works best. The parameter *kl* and parameter *kd* are selected in [1, 10, 20, 30, 40, 50], and the result is shown in Table 1. It can be observed that when *kl* is 20 and *kd* is 10, ResGCN-A has the best performance. After all parameters are adjusted, we calculated the 95% confidence interval of the model AUC values, which is (0.99124, 0.99164).

**Table 1:** The AUC value corresponds to different *kl* and *kd*.

*AUC*						
*kd*	*kl*					
	1	10	20	30	40	50
1	0.9821	0.9849	0.9852	0.9855	0.9850	0.9840
10	0.9897	0.9908	0.9916	0.9908	0.9904	0.9905
20	0.9905	0.9909	0.9912	0.9907	0.9903	0.9909
30	0.9902	0.9906	0.9904	0.9906	0.9906	0.9905
40	0.9887	0.9903	0.9903	0.9899	0.9900	0.9905
50	0.9892	0.9904	0.9902	0.9902	0.9899	0.9899

The experiment was carried out in the pytorch environment. The hardware environment is NVIDIA Quadro P620, with 5.8 G memory, and the CPU is Intel Core i7-9700. The time of training the ResGCN-A 1000 epoch is about 76 s.

### Selection of the number of GCN layers

**Figure 6:**
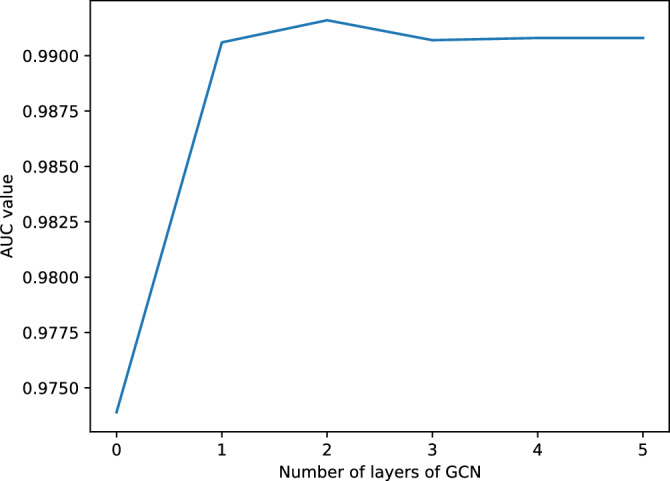
Different numbers of GCN layers.

In our model, the number of layers of GCN has a large impact on its performance. To achieve the best performance for our model, we set the number of layers of GCN to 0 to 5 respectively, and then performed 5-fold validation. The validation results are shown in Fig. 6. It can be observed that our model performs best when the number of layers of GCN is set to 2.

### The choice of classifiers

**Figure 7:**
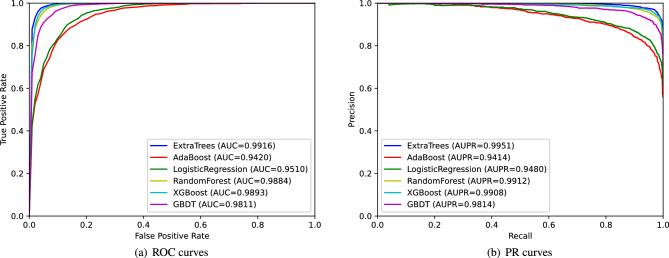
ROC curves and PR curves of different classifiers under 5-fold cross-validation.

We comprehensively evaluated the performance of six classifiers: Extra-Trees (extremely randomized trees)^26^, AdaBoost (adaptive Boosting)^27^, logistic regression^28^, random forest^29^, XGBoost (eXtreme Gradient Boosting)^30^ and GBDT (gradient boosting decision tree)^31^. Based on their AUC values and AUPR values obtained in the 5-fold cross-validation, the best-performing classifier is selected. The results are shown in Fig. 7a,b, with Extra-Trees having the highest AUC value and AUPR value. So we chose Extra-Trees as the classifier for ResGCN-A to predict potential lncRNA-disease associations.

### Attention mechanism verification

**Figure 8:**
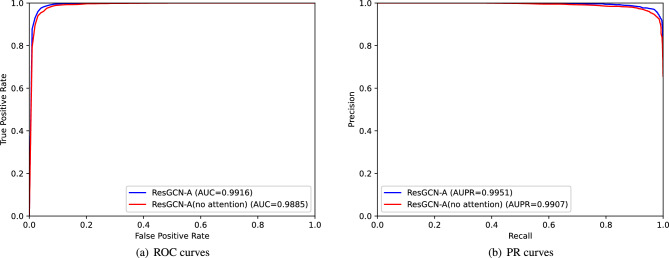
ROC curves and PR curves of ResGCN-A with and without attention mechanism under 5-fold cross-validation.

To verify the effectiveness of our proposed attention mechanism, we deleted the attention mechanism and adjusted the parameters of the model. Finally, when the learning rate is 0.001, the parameter *C* is 128, the *kl* is 20, and the *kd* is 10, we got the best result under the 5-fold cross-validation of the model. We compared the results with those of the original model. The comparison results are shown in Fig. 8a,b. By comparison, the AUC and AUPR values of the model are increased by $0.31\%$ and $0.44\%$ respectively by the attention mechanism, which indicates that the proposed attention mechanism can improve the prediction accuracy of the model.

### Robustness evaluation

**Figure 9:**
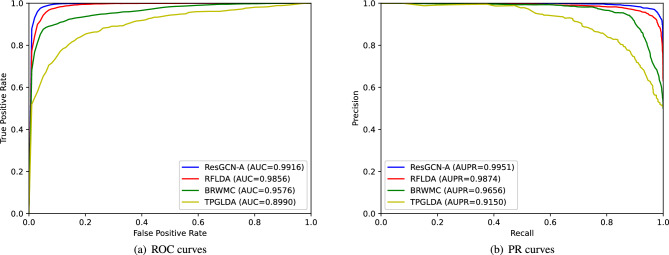
ROC curves and PR curves of ResGCN-A on different datasets under 5-fold cross-validation.

To evaluate the robustness of ResGCN-A, we kept the model parameters unchanged and evaluated it using 5-fold cross-validation on different data sets. Different data sets are from RFLDA^32^, BRWMC^33^, and TPGLDA^34^. The RFLDA dataset contains 240 lncRNAs, 495 miRNAs, 412 diseases, and 2697 experimentally validated lncRNA-disease associations. The BRWMC dataset contains 828 lncRNAs, 314 diseases, and 1695 lncRNA-disease associations. The TPGLDA dataset contains 115 lncRNAs, 178 diseases, 1415 genes, and 540 lncRNA-disease associations. We carried out 5-fold cross-validation of ResGCN-A under the data set in this paper, RFLDA data set, BRWMC data set and TPGLDA data set respectively. The experimental results are shown in Fig. 9a,b.

It can be seen that the AUC values and AUPR values of the model change slightly on RFLDA and BRWMC data sets, but the AUC values and AUPR values on TPGLDA data sets are lower. The reason is that the number of lncRNA-disease associations in the TPGLDA dataset is too small, and the data features cannot be fully utilized when training the model, resulting in low results. Therefore, we can conclude that increasing the number of known lncRNA-disease associations can improve the predictive performance of the model. Moreover, ResGCN-A has better robustness on data sets with a large number of associations.

### Comparison with other methods

In order to evaluate the performance of ResGCN-A, we compare it with GANLDA^12^, GAMCLDA^10^, GCRFLDA^35^, LDA-LNSUBRW^36^ and LDICDL^37^ using 5-fold cross-validation.

**Figure 10:**
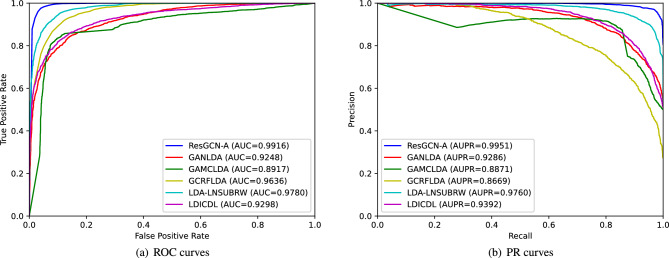
ROC curves and PR curves of ResGCN-A, GANLDA, GAMCLDA, GCRFLDA, LDA-LNSUBRW and LDICDL under 5-fold cross-validation.

As shown in Fig. 10a, the AUC value of ResGCN-A is 0.9916, which is higher than that of other methods (GANLDA 0.9248, GAMCLDA 0.8917, GCRFLDA 0.9636, LDA-LNSUBRW 0.9780 and LDICDL 0.9298). As shown in Fig. 10b, the AUPR value of ResGCN-A is 0.9951, which is better than that of other methods (GANLDA 0.9286, GAMCLDA 0.8871, GCRFLDA 0.8669, LDA-LNSUBRW 0.9760 and LDICDL 0.9392). The AUC and AUPR values of ResGCN-A are higher than those of other methods. The possible reason is that ResGCN-A adopts the method of combining residual graph convolutional network with attention mechanism to extract both local information and global information of original features, realizing the fusion of the two pieces of information and thus improving the prediction accuracy. However, there are some limitations to our approach. First, the method requires positive and negative samples to train the classifier, but it is still difficult to obtain reliable negative samples. The method of randomly selecting negative samples will affect the performance of the prediction method. In addition, compared with GANLDA and GAMCLDA, our method only collects the information of two biological entities, lncRNA and disease, and the features obtained are relatively one-sided. If more information of biological entities, such as miRNA and genes, can be added in the future, the features obtained will be more complete, which will also be of great help to the performance of the prediction method.

### Association prediction of diseases without associated lncRNA

It is important for prediction methods to have the ability to predict the associated lncRNAs for a given disease without any association. To evaluate the performance of our method for predicting diseases without associated lncRNA, we randomly selected ten diseases in the dataset. They are colon cancer, prostate cancer, gastric adenocarcinoma, lung cancer, liver cancer, breast cancer, HIV, kidney cancer, ovarian cancer and pancreatic cancer. For disease *d*, the number of associations it has is *k*. We firstly removed all lncRNAs associated with *d* and then used our model to predict lncRNAs associated with disease *d*. We counted the top *k* lncRNAs and the top $k+10$ lncRNAs predicted by the model under the two datasets respectively, and finally used Pubmed to search relevant literature to count the number of associations successfully predicted by our model. The statistical results are shown in Table 2. It can be observed that for liver cancer, there are 40 known associations, 40 of the top 40 lncRNAs predicted by the model are correct, and 45 of the top 50 lncRNAs predicted by the model are correct. The number of correct associations predicted by our model catches up with or even exceeds the number of original associations in the dataset, which indicates that our model has good predictive performance for diseases without associated lncRNA.

**Table 2:** Statistical results for diseases not associated with lncRNA.

Disease name	The number of confirmed associations(*k*)	Top *k*	Top $k+10$
Colon cancer	7	7	15
Prostate cancer	27	27	31
Gastric adenocarcinoma	3	3	8
Lung cancer	28	28	33
Liver cancer	40	40	45
Breast cancer	29	22	28
HIV	2	2	7
Kidney cancer	13	13	20
Ovarian cancer	16	14	16
Pancreatic cancer	12	11	17

### Case study

To further demonstrate the predictive ability of ResGCN-A for potential lncRNA-disease associations, case studies were conducted using prostate cancer and colon cancer as examples. In the case study, all known lncRNA-disease associations were taken as positive samples, unlabeled samples with the same number of positive samples were randomly selected as negative samples, and all positive and negative samples were taken as training sets. Unlabeled samples were taken as test sets and new lncRNA-disease associations were predicted by trained ResGCN-A. We selected the top 10 lncRNAs predicted by ResGCN-A to be associated with these two diseases, and the ten lncRNA associations with each disease were not confirmed in the original dataset. We used Pubmed to search for relevant literature to verify the associations predicted by our model.

Prostate cancer is an epithelial malignancy of the prostate gland, second only to lung cancer in male incidence. If the cancer can be detected at an early stage, the success rate of treatment is high. Table 3 shows the top 10 lncRNAs predicted by ResGCN-A to be associated with prostate cancer. Of those, 7 associations have been confirmed. It has been proven that high expression levels of the top-ranked XIST can lead to enhanced carcinogenicity of prostate cancer^38^. It has been shown that the third-ranked CCAT1 is promoted to overexpression in prostate cancer tumors that can lead to drug resistance^39^. It has been demonstrated that the fourth-ranked MIR17HG is a host gene of the miR-17-92a-1 cluster, and that when celastrol down-regulates androgen receptor and its target miR-17-92a, autophagy induction in prostate cancer cells is caused^40^. It has been confirmed that AFAP1-AS1, ranked 5th, can promote the metastasis of prostate cancer cells^41^. It has been shown that downregulation of the seventh-ranked ZFAS1 reduces the metastasis of prostate cancer cells^42^. It has been indicated that the 8th-ranked CCAT2 can downregulate miR-424 expression in prostate cancer^43^. It has been found that the 9th-ranked SOX2-OT can accelerate the proliferation and migration of prostate cancer cells via the miR-369-3p/CFL2 axis^44^.

**Table 3:** The top 10 lncRNAs associated with prostate cancer predicted by ResGCN-A that were not confirmed in the dataset.

Rank	LncRNA	Evidence
1	XIST	PMID: 16261845, 29212233, 36038831
2	TDRG1	Unknown
3	CCAT1	PMID: 29863242, 30221381, 31387890, 32089062, 34319909, 35773731
4	MIR17HG	PMID: 26891588, 27501757
5	AFAP1-AS1	PMID: 31081081, 31669642, 33138677, 33464230, 34126893
6	LSINCT5	Unknown
7	ZFAS1	PMID: 29416676, 31321444, 32104094, 36514044
8	CCAT2	PMID: 27558961, 31966650, 32218194, 32831916, 35178357, 37178445
9	SOX2-OT	PMID: 31623830, 32407168
10	MIR503HG	Unknown

Colon cancer is a common gastrointestinal malignant tumor occurring in the colon, which occupies the third place in the incidence rate of gastrointestinal tumors. Studying the lncRNA associated with colon cancer may be helpful for the prevention and treatment of colon cancer. Table 4 shows the top 10 lncRNAs predicted by ResGCN-A associated with colon cancer. Of these, 8 associations have been confirmed. It has been reported that NEAT1, which ranks first, can be involved in the occurrence and development of colon cancer^45^. It has been demonstrated that PVT1, which ranks second, was highly expressed in colon cancer tissue^46^. It has been confirmed that CDKN2B-AS1, ranked third, is an aberrant expression in human colorectal cancer^47^. It has been shown that the fourth-ranked GAS5 is down-regulated in colon cancer cell lines that are resistant to 5-fluorouracil^48^. It has been indicated that UCA1, ranked fifth, is a potential target for the treatment of colon cancer^49^. It has been confirmed that AFAP1-AS1, ranked sixth, can be used as a biomarker for the diagnosis and prognosis of patients with colorectal cancer^50^. It has been discovered that high expression of the seventh-ranked XIST is associated with reduced colorectal tumor growth^51^. It has been described that knockdown of HOTTIP, ranked eighth, inhibits the proliferation and migration of colorectal cancer cells^52^.

**Table 4:** The top 10 lncRNAs associated with colon cancer predicted by ResGCN-A that were not confirmed in the dataset.

Rank	LncRNA	Evidence
1	NEAT1	PMID: 31173354, 32077635, 32913527, 34055978, 34238666, 35676702
2	PVT1	PMID: 29552759, 30504754, 30820968, 34336125, 35763386, 35814832
3	CDKN2B-AS1	PMID: 34436551
4	GAS5	PMID: 27863421, 33416133, 36672566
5	UCA1	PMID: 30652355, 31989667, 32523353
6	AFAP1-AS1	PMID: 30588252, 31132513, 35937710
7	XIST	PMID: 29679755, 36116139
8	HOTTIP	PMID: 29274585
9	HOTAIRM1	Unknown
10	CAHM	Unknown

## Discussion

There is increasing evidence that lncRNA plays an important role in disease progression, and identifying disease-associated lncRNAs will contribute to a deeper understanding of disease mechanisms at the genetic level, which is of great significance for the prognosis, diagnosis and treatment of diseases. However, due to the complex associations between lncRNA and disease, and the long research time of traditional biological experiments, the cost of studying lncRNA-disease associations is still relatively high. To reduce the research cost of traditional biological experiments and improve the prediction accuracy of current computational methods, we proposed a lncRNA-disease associations prediction method with attention mechanism, ResGCN-A. It overcomes the problem that the previous methods can not make full use of the local and global information of the original features. ResGCN-A firstly integrated the two lncRNA similarity matrices and two disease similarity matrices respectively, and obtained the integrated lncRNA similarity matrix *lncSM* and the integrated disease similarity matrix *disSM*. Then, residual GCN was used to obtain the local feature information of *lncSM* and *disSM*. Then, the attention mechanism was used to obtain the global feature information of both. After batch normalization, the lncRNA potential feature *Lp* and the disease potential feature *Dp* were obtained. Finally, by concatenating the potential features, the training set of the Extra-Trees classifier was obtained, and the predicted lncRNA-disease associations were obtained by the trained Extra-Trees classifier. The performance of ResGCN-A was compared with the other five methods by 5-fold cross-validation. The results show that the AUC and AUPR values of ResGCN-A are higher than the corresponding values of the other five methods, so ResGCN-A is superior to the other five methods. Case studies showed that ResGCN-A was an effective method for predicting lncRNA-disease associations.

Although ResGCN-A has achieved good performances in the experiment, there are still some shortcomings that can be improved. There are three main points :(1) increasing the number of similarities in the original data. In this experiment, only four types of lncRNA Gaussian interaction profile kernel similarity, lncRNA functional similarity, disease Gaussian interaction profile kernel similarity and disease semantic similarity were used. In the future, more data similarity can be added to improve the prediction accuracy of this method, such as lncRNA sequence similarity. (2) Increasing the number of known lncRNA-disease associations. The robustness evaluation in this paper shows that increasing the number of known lncRNA-disease associations can improve the predictive performance of the model. In the future, more and more lncRNA-disease associations can be collected to improve the dataset. (3) Obtaining more reliable negative samples. Random selection of negative samples will affect the classification ability of the classifier. In the future, we hope to propose a more reliable negative sample selection method, which can improve the capability of the classifier and thus improve the accuracy of the prediction method.

## Data Availability

The datasets generated and/or analysed during the current study are available from the corresponding author upon reasonable request.
